# Supplementary material for: Validity of the Addiction-like Eating Behavior Scale among Patients with Compulsive Eating
Source: Nutrients. 2024 Sep 2;16(17):2932. doi: 10.3390/nu16172932 (PMC11396783; doi:10.3390/nu16172932)
Supplement: Supplementary file 1 [file nutrients-16-02932-s001.zip › nutrients-3169239-supplementary.pdf]

Table S1. Prediction of compulsive eating using AEBS items.

	t test	*β*	*F* test	R^2 change
**BES score**				
AEBS4 = I binge eat.	5.50, p < 0.001	0.31	124.51, p < 0.001	0.37
AEBS9 = I eat until I feel sick.	4.62, p < 0.001	0.25	43.92, p < 0.001	0.11
AEBS5 = When it comes to food, I tend to over-indulge.	3.79, p < 0.001	0.21	22.66, p < 0.001	0.05
AEBS8 = Despite trying to eat healthy, I end up eating 'naughty' foods.	2.61, p = 0.01	0.15	10.13, p = 0.002	0.02
AEBS15 = I feel unable to control my weight.	2.24, p = 0.026	0.11	5.01, p = 0.026	0.01
**GQ score**				
AEBS4 = I binge eat.	5.02, p < 0.001	0.34	81.27, p < 0.001	0.27
AEBS5 = When it comes to food, I tend to over-indulge.	1.91, p = 0.050	0.14	13.77, p < 0.001	0.04
AEBS7 = Once I start eating certain foods, I can't stop until there's nothing left.	2.20, p = 0.029	0.15	5.34, p = 0.022	0.02
AEBS10 = I continue to eat certain unhealthy foods despite […] their effects on my health.	2.01, p = 0.046	0.12	4.04, p = 0.046	0.01
**FCQTr score**				
AEBS4 = I binge eat.	4.95, p < 0.001	0.26	118.11, p < 0.001	0.35
AEBS8 = Despite trying to eat healthy, I end up eating 'naughty' foods.	3.67, p < 0.001	0.21	62.87, p < 0.001	0.15
AEBS7 = Once I start eating certain foods, I can't stop until there's nothing left.	3.53, p < 0.001	0.20	26.89, p < 0.001	0.06
AEBS5 = When it comes to food, I tend to over-indulge.	3.33, p < 0.001	0.19	11.95, p < 0.001	0.02
AEBS12 = I don't eat a lot of high fat/sugar foods.	-2.85, p = 0.005	-0.15	7.80, p = 0.006	0.02
AEBS15 = I feel unable to control my weight.	2.42, p = 0.016	0.11	5.88, p = 0.016	0.01

Note. AEBS = Addiction-like Eating Behavior Scale; BES = Binge Eating Scale; GQ = Grazing Questionnaire; FCQTr = Food Cravings Questionnaire Trait reduced.

Table S2. Accuracy of the different AEBS cut-off scores to capture YFAS food addiction.

	**True +**	**True -**	**Correct**	**False +**	**False -**	**Errors**	**Sensitivity**	**Specificity**
**Score ≥ 39**	66.82	9.09	75.91	22.27	1.82	24.09	97.35	28.99
**Score ≥ 41**	64.55	10.91	75.45	20.45	4.09	24.55	94.04	34.78
**Score ≥ 46**	57.27	16.36	73.64	15.00	11.36	26.36	83.44	52.17
**Score ≥ 49**	48.18	20.91	69.09	10.45	20.45	30.91	70.20	66.67
**Score ≥ 55**	29.55	28.63	58.18	2.73	39.09	41.82	43.05	91.30

Note. AEBS = Addiction-like Eating Behavior Scale; YFAS = Yale Food Addiction Scale.
